# The role of γδ T cells in airway epithelial injury and bronchial responsiveness after chlorine gas exposure in mice

**DOI:** 10.1186/1465-9921-8-21

**Published:** 2007-03-07

**Authors:** Hossein Koohsari, Meiyo Tamaoka, Holly R Campbell, James G Martin

**Affiliations:** 1 Meakins-Christie Laboratories, McGill University, Montreal, QC, Canada

## Abstract

**Background:**

Acute exposure to chlorine (Cl_2_) gas causes epithelial injury and airway dysfunction. γδ T cells are present in the mucosal surface of the airways and may contribute to the injury/repair response of the epithelium.

**Methods:**

C57Bl/6J (wild type) and TCR-δ^-/- ^mice exposed to Cl_2 _(400 ppm) for 5 minutes underwent measurements of airway responses to i.v. methacholine (MCh) at 1, 3, and 5 days after exposure. Bronchoalveolar lavage was performed to determine epithelial and leukocyte counts, and protein content. Tissue repair was assessed by proliferating cell nuclear antigen (PCNA) immunoreactivity and by expression of keratinocyte growth factor (KGF) mRNA by real-time PCR.

**Results:**

Wild type mice developed a greater degree of airway hyperresponsiveness to MCh at 1 day post exposure to Cl_2 _compared with TCR-δ^-/- ^mice. Epithelial cell counts in BAL after Cl_2 _exposure were greater in TCR-δ^-/- ^mice, but macrophages showed a later peak and granulocyte numbers were lower in TCR-δ^-/- ^than in wild type mice. Both groups had increased levels of total protein content in BAL after Cl_2 _exposure that resolved after 3 and 5 days, respectively. Epithelial proliferating cell nuclear antigen staining was increased at 1 and 3 days post exposure and was similar in the two groups. KGF mRNA was constitutively expressed in both groups and did not increase significantly after Cl_2 _but expression was lower in TCR-δ^-/- ^mice.

**Conclusion:**

The severity of airway epithelial injury after Cl_2 _is greater in TCR-δ^-/- ^mice but the inflammatory response and the change in airway responsiveness to methacholine are reduced. The rates of epithelial regeneration are comparable in both groups.

## Background

Although chlorine exposures were first described in association with chemical warfare, currently most exposures are accidental in industries such as pulp and paper mills [1-3], in swimming pools due to release of Cl_2 _gas from chlorinators [4], and in the home where Cl_2 _gas can be released by mixing bleach with other cleaning products [5]. Effects on epithelial cell function may also be associated with chlorine in the swimming pool environment [6]. The effects of acute chlorine gas inhalation *in vivo *have been investigated in rodent and murine models [7,8]. High concentrations cause early airspace and interstitial edema associated with bronchial epithelial sloughing. There is mucosal infiltration by polymorphonuclear leukocytes, and subsequent epithelial regeneration, marked by epithelial hyperplasia and goblet cell metaplasia [7]. An additional feature of remodeling is an increase in airway smooth muscle mass [8]. Increased lung resistance and/or bronchial hyperresponsiveness to inhaled methacholine have also been observed [8]. Changes in lung function relate to the extent of airway epithelial damage and the degree of BAL neutrophilia [7].

A murine model (A/J strain) of irritant induced asthma caused by acute chlorine exposure [8] showed a significant increase in airway responsiveness and inflammation with 400–800 ppm Cl_2 _at 24 hours post-exposure that correlated with airway epithelial damage and shedding. Furthermore, this study provided evidence of oxidant stress and nitrosylation of proteins in airway epithelial cells and alveolar macrophages [8]. Porcine and rabbit models of Cl_2 _injury have demonstrated similar histological and lung function findings, where increases in pulmonary resistance and elastance, edema, sloughing of bronchial epithelium, and inflammatory cell influx were observed [9-11]. According to the standards set by the National Institute for Occupational Health and Safety (US) more than 30 ppm for an hour or more can cause substantial damage. The lowest reported fatal exposure was to a concentration of 430 ppm. The brief exposure employed in the current study is likely within the range of possible accidental exposures of human subjects.

The factors influencing the rate of epithelial regeneration are likely of key importance in determining the short and long term consequences of chlorine induced airway dysfunction. The γδ T cells are trophic for the epithelium and potentially could influence the regenerative response of the epithelium to chlorine [12]. To evaluate the role of γδ T cells in chlorine induced airway injury we studied the responses of TCR δ ^-/- ^(γδ T cell deficient) mice to a single exposure to chlorine. We hypothesized that γδ T-cells were involved in modulating airway responses to methacholine and the repair of airway epithelium after acute chlorine gas exposure. The γδ T cells express the epithelial cell mitogen keratinocyte growth factor (KGF) which again suggests that these cells may be involved in preventing damage or repairing damaged epithelial cells [13,14].

## Methods

### Animals

Male C57BL/6J and TCR δ -/- (B6.129P2-Tcrd tm1Mom) mice 8 to 10 weeks of age were purchased from Jackson Laboratories. All animals were housed in a conventional animal care facility at McGill University. All the experiments were approved by the Animal Care Committee of McGill University.

### Experimental protocol

Chlorine gas (Matheson Gas Products, Ottawa, Canada) was mixed with room air in a standard 3 L re-breathing bag to make a concentration of 400 ppm Cl_2_. The intake port of an exposure chamber was connected to the re-breathing bag while the outlet port was connected to a flow meter and vacuum. Animals were restrained to receive nose-only exposure for 5 minutes. In mice exposed to Cl_2 _lung function was evaluated 1, 3, and 5 days after exposure. The animals were assessed for airway responsiveness to methacholine (n = 8) and BAL leukocyte counts and immunohistochemical staining were performed (n = 7) on each of the test days.

### Evaluation of Airway Responsiveness

Mice were sedated with an intraperitoneal (i.p) injection of xylazine hydrochloride (8 mg/kg) and anaesthetized with pentobarbital (30 mg/kg) injected through a catheter placed in the left jugular vein. Subsequently, the animal was tracheostomized and was connected to a small animal ventilator (Flexivent, Scireq, Montreal, Canada). Muscle paralysis was induced with pancuronium bromide (0.2 mg/kg i.v.). The mice were ventilated in a quasi-sinusoidal fashion with 150 breaths/min, a tidal volume of 0.18 ml and a PEEP of 2–3 cm H_2_O. Methacholine (MCh) was administered via the jugular catheter in doubling doses ranging from 10 to 640 ug/kg. Respiratory system resistance (Rrs) and elastance (Edyn, rs) were determined before challenge and after each dose of MCh. The peak responses are reported.

### Bronchoalveolar Lavage Fluid Analysis

Following measurements of respiratory function the animals were killed with an overdose of sodium pentobarbital and were exsanguinated. The lungs were lavaged with 0.6 ml of sterile saline, followed by four aliquots of 1 ml each. The first aliquot of BAL fluid was centrifuged at 1600 rpm for 5 minutes at 4°C and the supernatant was retained for measurements of protein by Bradford assay. The cell pellet was pooled with the remaining lavage samples and total cell numbers were counted with a hemacytometer. The cytospin slides of BAL cells were stained with Dip Quick (Jorgensen Labs Inc., Loveland, CO). Differential cell counts were based on a count of 300 cells. Absolute cell numbers for individual leukocytes were also calculated as the product of the total and differential cell counts. Epithelial cells were identified by the ciliated border and their tendency to detach in clumps.

### Histology and immunohistochemistry

Following harvesting the lungs were perfused with saline until the effluent was clear. Subsequently lung tissues were fixed overnight with 10% formalin at a pressure of 25 cm of H_2_O. Formalin-fixed tissues were embedded in paraffin blocks, cut into 5 μm sections and placed on Superfrosst slides. To evaluate the repair response of the airway epithelial cells a specific mouse anti-proliferating cell nuclear antigen (PCNA) monoclonal antibody was used.

For immunohistochemical detection of PCNA, slides were deparaffinized with xylene and dehydrated with ethanol. Slides were placed in Antigen Unmasking Solution (Vector Laboratories, CA) and treated with high temperature antigen retrieval. Cells were permeabilized using 0.2% Triton X-100 detergent. A mouse-on-mouse kit was used to reveal PCNA immunoreactivity. Prior to application of primary mouse anti-PCNA antibody tissues were blocked using mouse IgG blocking reagent to reduce non-specific binding. The tissues were then treated with anti-PCNA antibody or isotype control antibody (negative control) for 30 minutes at 37°C, rinsed with TBS and treated with biotinylated anti-mouse IgG reagent. An avidin-biotin complex alkaline phosphatase (ABC-AP, Vectastain) kit followed by alkaline phosphatase substrate was used for development. Tissues were counterstained with methyl green. Mouse intestinal tissue was used as a positive control. Adjacent tissue sections were stained with hematoxylin and eosin for routine histological examination.

### Morphometry

For quantitative analysis of PCNA immunoreactivity, airways were traced using a camera lucida side arm attachment to the microscope (20× magnification) and the positively stained epithelial cells were counted. The airway images were then scanned (Canon, Lake Success, NY) and digitized using a digitizing tablet (Wacom, Vancouver, WA) and commercial software (Sigma Scan, Leesburg, VA) to calculate airway perimeter length. Results were then expressed as the number of PCNA positive cells/mm of basement membrane.

### RT and quantitative real-time PCR for KGF in the lung

The left lung was homogenized in Trizol Reagent^® ^(Invitrogen) and total RNA was extracted according to the manufacturer's instructions. 2 mg of RNA was reverse transcribed to cDNA with Superscript II (Invitrogen) and quantitative real-time PCR was performed using a LightCycler (Roche). The following pairs of primers were used for amplification; KGF: 5'-ACG AGG CAA AGT GAA AGG GA-3', 5'-TGC CAC AAT TCC AAC TGC CA-3', ribosomal protein S9: 5'-AAG CAA CTG ATT GAA CCC GTG CAG-3', 5'-ATC TTC CCG CTT CCG TGC TCA TAA-3'. The copy number was calculated based on the standard curves established for each growth factor and a housekeeping gene. Briefly, PCR products were extracted from agarose gel and purified with GFX PCR DNA and Gel Band Purification Kit (Amersham Biosciences). The amount of PCR product was calculated by densitometry. 10^1^–10^10 ^copies of standard were prepared by step dilution. The expression of KGF was standardized for S9 expression.

### Statistical analysis

Comparison among several means was done by analysis of variance and post hoc testing was done using Fisher least significant difference test. P-values less than 0.05 were considered significant.

## Results

### Changes in bronchoalveolar lavage composition after chlorine gas exposure

**Figure 1:**
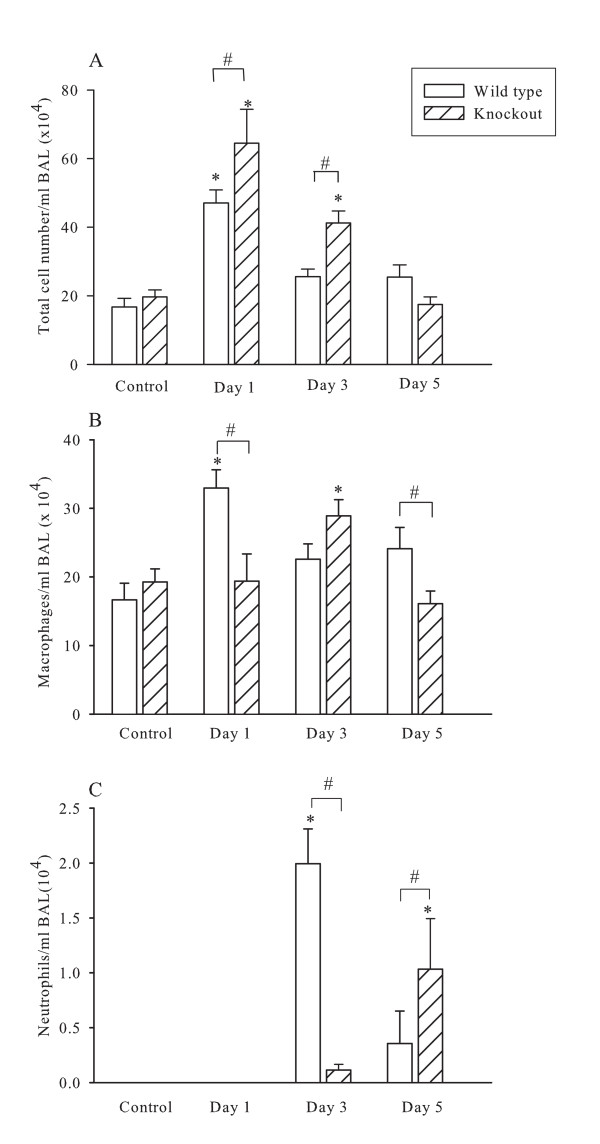
**Cellular composition of bronchoalveolar lavage**. Data for control and chlorine exposed animals that were sacrificed 1, 3 and 5 days after chlorine are shown. Both γδ T cell deficient mice and wild type animals are demonstrated. Panel A. Total cells recovered from bronchoalveolar lavage. Panel B Total macrophage cell counts in BAL fluid at baseline and at 1, 3 and 5 days after Cl_2 _exposure for knock out and wild type animals. Panel C. Neutrophil counts in BAL fluid. * P < 0.05 compared to 0 ppm control. # P < 0.05.

Bronchoalveolar lavage was performed at days 1, 3 and 5 after chlorine exposure. The fluid recovered by BAL averaged 85% of the volume instilled and did not differ significantly among the groups. Total cell counts were increased by 24 hours after exposure to chlorine and returned to baseline values after 3 days in wild type and 5 days in knockout mice (figure 1A). There was a marked difference in cell viability (trypan blue exclusion) among different groups and the difference was significant between wild type (49% non-viable) and knockout animals (59% non-viable; p < 0.05). Non-viable cells were principally epithelial cells. These values returned towards baseline at 3 days in wild type and at 5 days in knockout mice. The increase in total cell counts was mostly attributable to increases in macrophage numbers (figure 1B). However, there were also significant increases in neutrophils (Figure 1C). A delayed and lower macrophage and neutrophil influx into the BAL was observed in γδ T cell deficient mice. Macrophage numbers increased significantly in wild type compared to control mice 24 hrs after exposure; while a significant but transient increase was observed in knockout animals at 3 days post exposure (figure 1B). At the 5-day time point wild type mice still had a significantly larger number of macrophages in BAL compared to knockouts. The same pattern of cellular recruitment was observed for neutrophils (figure 1C) but the increase in neutrophil numbers was significant at 3 and 5 days for wild type and knockout mice, respectively.

**Figure 2:**
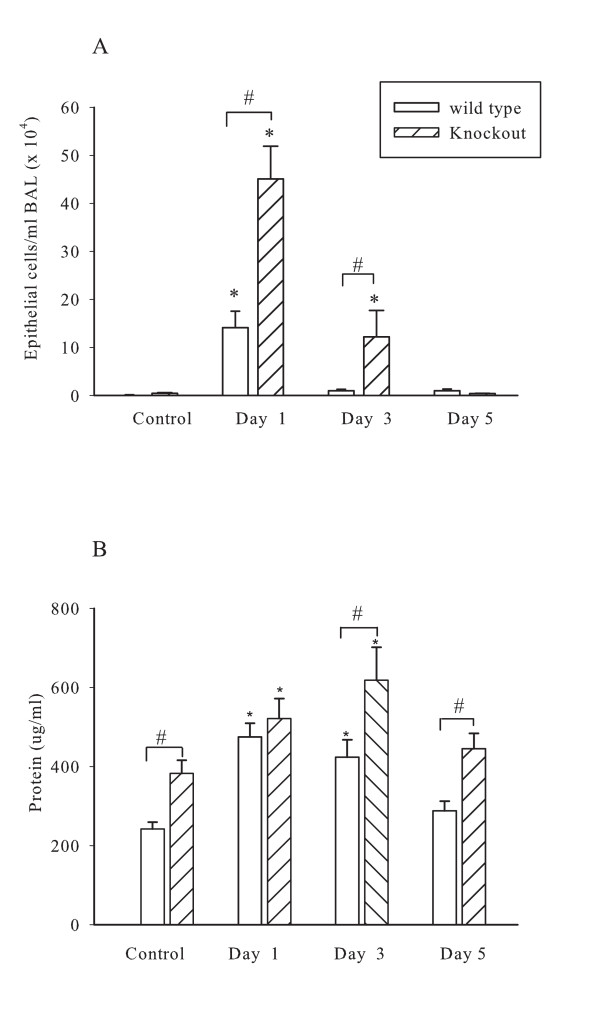
**Epithelial cell shedding and protein in bronchoalveolar lavage fluid after Cl_2 _gas exposure**. Panel A. Epithelial cell counts in BAL fluid. Panel B. Protein levels in BAL fluid, measured using a Bradford assay. * P < 0.05 compared to 0 ppm control. # P < 0.05.

To assess the extent of damage caused by inhalation of Cl_2 _gas, epithelial cell counts and BAL protein content were measured. Cl_2 _inhalation caused extensive shedding of the airway epithelial cells (figure 2A). A significant increase in the number of epithelial cells in BAL was observed 24 hrs after Cl_2 _exposure in both groups.

However, knockout mice appeared to be more susceptible to epithelial damage or shedding as evidenced by epithelial cell counts in BAL. Epithelial cells were cleared rapidly in wild type mice while knockout mice still had slightly elevated epithelial counts even at 3 days post exposure.

Total protein content in BAL supernatant was significantly greater at baseline in the γδ T cell deficient mice than the control wild type animals. The BAL protein was significantly elevated at days 1 and 3 after exposure to Cl_2 _in both groups and was higher in the γδ T cell deficient mice at day 3. BAL protein returned to baseline values by day 5 although it was still significantly higher in knockout animals (Figure 2B).

### Histologic and immunohistochemical findings after chlorine gas exposure

The airways of animals exposed to Cl_2 _gas showed marked epithelial loss and replacement of the cuboidal ciliated epithelium with flat cells. Knockout mice exposed to Cl_2 _also sustained damage to the tissue around the airways at the 24 hour time point. Accumulation of inflammatory cells in alveolar walls was also observed. There were no obvious differences in lung histology between wild type and knockout animals prior to exposure to Cl_2_.

**Figure 3:**
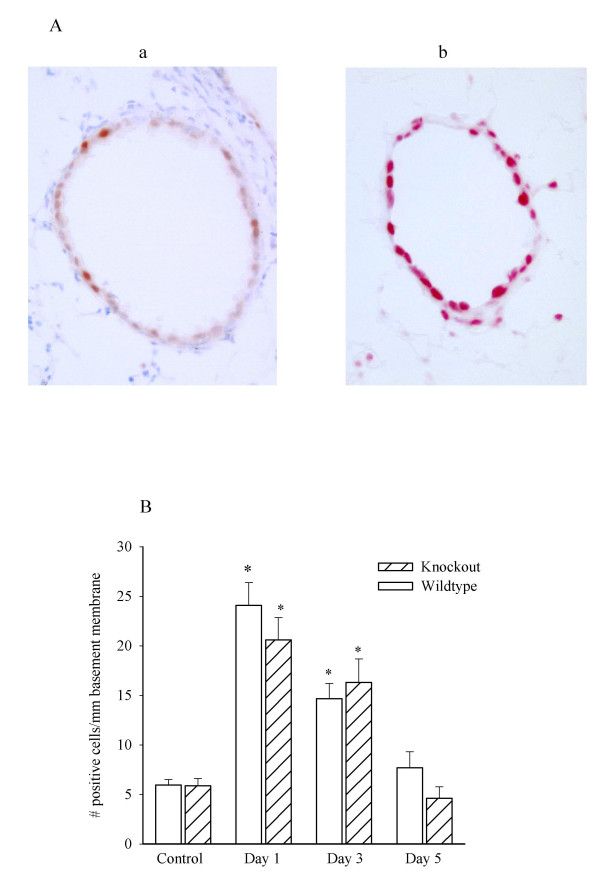
**Effects of chlorine on epithelial cell proliferation. **Panel A. Representative pictures showing PCNA immunostaining in airway epithelial cells before (a) and 1 day after exposure to 400 ppm Cl_2 _gas (b) in wild type mice. Panel B. Numbers of epithelial cells with positive staining for PCNA per mm of basement membrane. Knockout mice have impaired epithelial cell regeneration following Cl_2 _gas injury. The vertical bars indicate one SEM. * P < 0.05 compared to 0 ppm control.

Epithelial regeneration was evaluated by assessing PCNA-positive epithelial cells (Figure 3A). Quantitative analysis of the PCNA immunoreactivity in the epithelium showed no difference between wild type and knockout control animals under baseline conditions (Figure 3B). At the 24 h time point following a 5 minute exposure to 400 ppm Cl_2 _there was a significant increase in epithelial cell proliferation in both groups. The knockouts seemed comparable in the rate of regeneration of epithelium compared to the wild type animals, with the exception of a slightly lower signal at 1 and 5 days.

The regenerative response was sustained in wild type animals for up to 3 days. Both groups returned to baseline numbers of PCNA positive cells by five days after initial Cl_2 _injury.

### Effects of chlorine exposure on bronchial responsiveness

**Figure 4:**
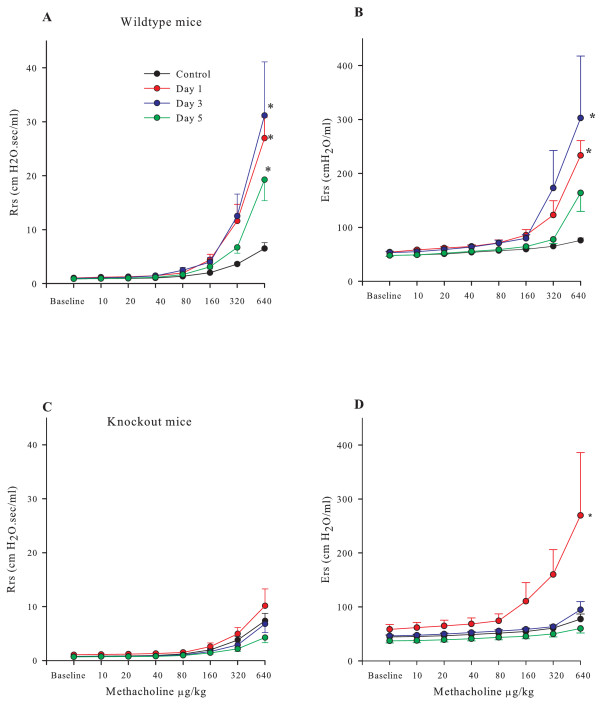
**Methacholine responsiveness after chlorine exposure. **The values of respiratory system resistance (A; R_RS_) and respiratory system dynamic elastance (B; E_RS_) following intravenous injection of methacholine in wildtype and knockout mice. The values of respiratory system resistance (C; R_RS_) and respiratory system dynamic elastance (D; E_RS_) in TCR δ knockout mice are shown. The vertical bars indicate one SEM. * P < 0.05 compared to 0 ppm control.

The airway responsiveness to methacholine in the mice exposed to 400 ppm Cl_2 _was examined also at 1, 3, and 5 days after exposure. There were no baseline differences in Rrs and Ers between wild type and knockout mice and between sham-exposed and Cl_2 _exposed groups (Figure 4A and 4B). Wild type mice had a significant increase in methacholine responsiveness compared at 1 day after exposure to 400 ppm Cl_2_^. ^Although the degree of responsiveness decreased slightly by day 5, it was still significantly elevated compared to sham-exposed controls (Figures 4A and 4B). Knockout mice did not develop significant AHR to methacholine at any of the time points, with the exception of a transient increase in methacholine-induced change in Ers 1 day after exposure (figure 4C and 4D).

### Effects of chlorine on keratinocyte growth factor expression

**Figure 5:**
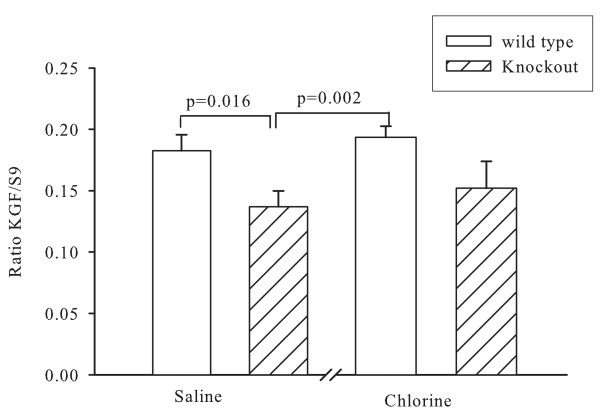
**mRNA for keratinocyte growth factor in lungs following chlorine exposure. **KGF mRNA expression was assessed by real-time PCR and was referenced to the levels of the housekeeping gene S9.

KGF mRNA expression was assessed by real-time PCR. There was constitutive expression in both wild type and knockout animals and the level of expression corrected for the house-keeping gene S9 was greater in the former animals (p = 0.016). There was no significant increase in expression following Cl_2 _exposure in either group (Figure 5).

## Discussion

In this study we examined the injury and repair response of mice to acute exposure with 400 ppm Cl_2 _gas, a highly reactive gas implicated in irritant induced asthma. Our findings indicate that the response to airway injury with chlorine differs between wild type and γδ T cell deficient mice. Wild type mice have more inflammation but a comparable rate of epithelial regeneration compared to γδ T cell deficient mice. Interestingly the airway responsiveness to methacholine increased in the wild type but not the knockout mice after chlorine exposure, consistent with the difference in the magnitude of the inflammatory response in the two study groups. Differences in levels of constitutive expression of KGF do not seem to play a substantial role in determining the rates of epithelial cell proliferation.

By 24 hours after chlorine exposure bronchoalveolar lavage fluid analysis showed increased protein content in the airways, which is likely attributable to microvascular leak and cellular necrosis. Indeed there were increases in the numbers of shed epithelial cells and histological evidence of epithelial denudation. Epithelial cell regeneration, as evidenced by PCNA immunoreactivity, was relatively rapid in wild type animals and returned to baseline after 5 days. The epithelial proliferative response in γδ T cell deficient mice was slightly less at 1 and 5 days post exposure than in wild type mice despite the shedding of greater numbers of epithelial cells.. Direct oxidative stress or damage secondary to neutrophil activation could contribute to the extent of shedding [15]. The latter mechanism seems less likely since the inflammatory response to epithelial damage was also attenuated in the γδ T cell deficient mice. Our findings are consistent with a role of γδ T cells in determining the magnitude of the inflammatory response to acute epithelial injury and in maintaining and repairing the epithelial barrier [16]. Chen et al. have found that a deficiency of γδ T cells rendered the intestinal epithelium of mice more susceptible to dextran sodium sulphate (DSS) induced colitis [14]. A similarly reduced response to epithelial injury in this model was attributed to a lack of KGF production by γδ T cells. Similar roles for these cells in wound repair have been shown [17]. Although it seemed *a priori *highly likely that similar mechanisms were involved in the repair of the bronchial epithelium there are significant differences in γδ T cell distribution in epidermis and bronchial epithelium. The γδ T cells are relatively uncommon, representing less than 10% of the total T cells in the lung and are described as being virtually absent from the bronchial epithelium itself [18]. This observation is consistent with the finding that differences in epithelial repair resulting from γδ T cell deficiency in the airways are minor and may be less than in other epithelial tissues.

Wild type mice demonstrated AHR following Cl_2 _exposure that was still present 5 days later. However the γδ T cell deficient mice developed a mild degree of AHR at 1 day after Cl_2 _exposure that was detected by changes in elastance only. This response suggests that a more peripheral pulmonary response may have occurred in the knockout mice, because resistance is more reflective of central and peripheral pulmonary responses. The difference in the degree of AHR between knock out and wild type animals is more closely associated with the intensity of inflammation which was greater in wild type animals and not epithelial shedding which was greater in the knockout group. The loss of epithelial nitric oxide or dilator prostaglandins could potentially affect airway responsiveness but these factors seem improbable causes of AHR because epithelial shedding was in fact greater in knockout animals. Differences in the intensity of inflammation between groups are more likely to be the explanation. The mechanism of AHR following Cl_2 _may be similar to that of ozone in that both forms of injury are associated with oxidant damage to the tissues. There appear to some differences in the clinical consequences of the injuries but there are also substantial similarities [19]. Neutrophilic inflammation is associated with oxidant gas exposures and has been shown in the dog to be important for the development of AHR [20]. Indeed AHR after ozone exposure is reduced by neutrophil depletion [21]. The more marked inflammation in wild type animals in the current study is consistent with these findings.

Other factors could account for chlorine induced AHR in wild type mice. Epithelial cell swelling has been argued to be a significant contributor to AHR following allergen challenge in the mouse through its encroachment on the airway lumen [21]. Whether such an effect occurs after chlorine in mice is not known. Airway instability or, otherwise stated, the tendency of the airway to close may also cause AHR in the mouse [22]. Following allergen challenge, and presumably other pro-inflammatory stimuli, the disruption of airway surfactant function by fibrin contributes to the observed AHR [23]. Cl_2 _exposure increased bronchoalveolar lavage protein to a greater extent in wild type animals consistent with a role for airway protein in airway dysfunction. However peak protein levels were comparable in both groups because of baseline differences in protein in the airways of knockout animals, so that it is difficult to conclude that protein induced changes in airway stability and closure contributed to AHR in the current study. We speculate that the increases in BAL protein levels in γδ T cell deficient mice under baseline conditions indicate compromise of the epithelial barrier. Similar findings have been reported for the epidermis of γδ T cell deficient mice which demonstrates abnormal electrical impedance, indicative of susceptibility to dehydration [24].

The recruitment of phagocytic cells is an important mechanism for removal of damaged epithelial cells [25]. Increases in neutrophils and macrophages were greater in wild type mice whereas shed epithelial cells are more numerous in γδ T cell deficient mice, suggesting more epithelial damage but less inflammation in the γδ T cell deficient mice. The inflammation, also affecting macrophages and neutrophils, in response to epithelial necrosis induced by ozone exposure has been shown previously to be muted in γδ T cell deficient mice [26]. The explanation for the reduced inflammatory response is unclear but the close proximity of γδ T cells and macrophages and dendritic cells in the airways provides pathways by which inflammation could be affected [18].

In summary γδ T cell deficient mice have high numbers of epithelial cells in bronchoalveolar lavage fluid, indicating greater epithelial injury following chlorine exposure. However epithelial cell regeneration was comparable in the two groups. The γδ T cell deficient mice also had an attenuated inflammatory response compared to wild type mice. The lack of γδ T cells was associated with an abrogation of the changes in responsiveness to methacholine, suggesting that the intensity of the inflammatory response may be responsible for this phenomenon. These conclusions are tentative, based on associations which do not necessarily indicate cause and effect relationships and therefore will require confirmation.

## Conclusion

Chlorine causes airway injury associated with increase in airway responsiveness to methacholine and airway inflammation. γδ T cell deficient mice shed more epithelial cells but have no airway hyperresponsiveness and exhibit an attenuated inflammatory response. The contribution of γδ T cells to epithelial regeneration in the intestine is not evident in the airways.

## Competing interests

The author(s) declare that they have no competing interests.

## Authors' contributions

HK performed all the experiments and data analysis.

MT performed the real-time PCR for keratinocyte growth factor.

HRC assisted in the performance of the measurements of responsiveness to methacholine.

JGM designed the study, supervised the experimental work and wrote the final manuscript. All authors read and approved the final manuscript.
